# HL156A, a novel pharmacological agent with potent adenosine-monophosphate-activated protein kinase (AMPK) activator activity ameliorates renal fibrosis in a rat unilateral ureteral obstruction model

**DOI:** 10.1371/journal.pone.0201692

**Published:** 2018-08-30

**Authors:** Bodokhsuren Tsogbadrakh, Kyung Don Ju, Jinho Lee, Miyeun Han, Junga Koh, Yeonsil Yu, Hajeong Lee, Kyung-Sang Yu, Yun Kyu Oh, Hyo Jin Kim, Curie Ahn, Kook-Hwan Oh

**Affiliations:** 1 Biomedical Research Institute, Seoul National University Hospital, Seoul, Korea; 2 Department of Internal Medicine, Seoul National University College of Medicine, Seoul, Korea; 3 Renal Division, Department of Internal Medicine, Seoul National University Hospital, Seoul, Korea; 4 Department of Clinical Pharmacology and Therapeutics, Seoul National University College of Medicine and Hospital, Seoul, Korea; 5 Department of Internal Medicine, Seoul National University Boramae Medical Center, Seoul, Korea; 6 Department of Internal Medicine, Dongkuk University, Kyungju, Korea; 7 Transplantation Research Institute, Seoul National University Hospital, Seoul, Korea; Hopital Tenon, FRANCE

## Abstract

**Background:**

Renal fibrosis is characterized by excessive production and deposition of extracellular matrix (ECM), which leads to progressive renal failure. Adenosine-monophosphate-activated protein kinase (AMPK) is a highly conserved kinase that plays a key role in Smad-3 signaling. Here, we examined the effect of a novel AMPK activator, HL156A, on the inhibition of renal fibrosis in *in vivo* and *in vitro* models.

**Methods:**

Unilateral ureteral obstruction (UUO) was induced in male Wistar rats. Rats with UUO were administered HL156A (20mg/kg/day), and then the kidneys were harvested 10 days after ligation for further analysis.

**Results:**

In the rat UUO model, HL156A attenuated ECM protein deposition. After HL156A treatment, expressions of TGF-β1, p-Smad3, α-SMA, fibronectin, and type IV collagen were suppressed, and E-cadherin expression was up-regulated. In the *in vitro* experiment, NRK52E cells were treated with HL156A before TGF-β1 stimulation. The inhibitory effects of HL156A upon the signaling pathways and markers of the epithelial-to-mesenchymal transition (EMT) were analyzed. In TGF-β1-treated NRK-52E cells, HL156A co-treatment inhibited the TGF-β1-induced Smad3 signaling pathway and EMT markers.

**Conclusion:**

Taken together, the above findings suggest that HL156A, a novel AMPK activator, ameliorates renal fibrosis *in vivo* and *in vitro*.

## Introduction

Renal fibrosis, characterized by glomerulosclerosis and tubulointerstitial fibrosis, is the final common pathway of many kidney diseases[1]. Tubulointerstitial fibrosis is associated with epithelial-mesenchymal transition (EMT)[2], and synthesis of extracellular matrix (ECM). ECM components, including type I and IV collagen and fibronectin, are the hallmarks of tubulointerstitial fibrosis[3]. Transforming growth factor-β (TGF-β) signaling is one of the most important pathways associated with renal fibrosis by activating its downstream Smad signaling pathway[4], [5], [6]. TGF-β1 is reportedly up-regulated in response to injurious stimuli, such as unilateral ureteral obstruction (UUO) [7]. UUO is a well-known model of renal fibrosis[8], [9]. UUO causes renal metabolic changes, leading to tubular injury and renal inflammation, characterized by macrophage infiltration[10].

Adenosine monophosphate protein kinase (AMPK) in a sensor of cellular energy storage and a powerful metabolic regulator. AMPK is a heterotrimeric protein consisting of α1/2, β1/2, and γ1/2/3 subunits and is a sensor of cellular energy status[11]. AMPK activation requires phosphorylation of Thr172 on the “loop activation” of the α-subunits[12]. AMP-activated AMPK is a major cellular regulator of glucose and lipid metabolism[13], [14]. The biological consequences of AMPK activation are diverse and include glucose and lipid metabolism[15], cytokine production and inflammation regulation[16], and cell proliferation and apoptosis[17]. Metformin, an AMPK activator, is widely used to treat type-2 diabetes [18]. Furthermore, recent studies have shown that AMPK activation by metformin inhibited albumin-induced ER stress in tubular epithelial cells in a rat model [19], [20]. The activation of AMPK by metformin has been applied in clinical practice to treat type 2 diabetes mellitus and renal fibrosis[21]. In diabetic rats, metformin and AICAR increased renal AMPK phosphorylation, inhibited mTOR activation, and attenuated renal hypertrophy without affecting hyperglycemia [18]. HL156A (Hanall Pharmaceuticals Co. Ltd, Seoul, Korea) is a recently developed molecule that is a more potent AMPK activator than AICAR or metformin. In a previous study, we showed that HL156A had a protective effect against peritoneal fibrosis (PF)[22]. In this study, we examined the anti-fibrotic effect of AMPK activation by HL156A in both *in vivo* and *in vitro* renal fibrosis models.

## Materials and methods

### AMPK activity

The level of activated AMPK in NRK-52E cells (ATCC, Manassas, VA, USA), a renal proximal tubular cell line, was determined using an ELISA kit (KHO0651; Invitrogen Corporation, Camarillo, CA, USA) to measure AMPK-α phosphorylation at Thr172, following the manufacturer’s instructions. To compare the activity of the three AMPK activators, NRK-52E cells were treated with 5-aminoimidazole-4-carboxamide-1-^®^-D-ribofuranoside (AICAR, sc-200659A; Santa Cruz Biotechnology, CA, USA), metformin (1115-70-4; Sigma-Aldrich St. Louis, MO), and HL156A (Hanall Pharmaceuticals, Seoul, Korea) at 0, 10, 30, and 50 mM, respectively. Detailed methods for generating HL156A have been described elsewhere [22].

### Animal model of unilateral ureteral obstruction

All animal experiments were performed with the approval of the Institutional Animal Care and Use Committee (IACUC-13-0211) of Seoul National University Hospital. First, we performed a preliminary experiment in order to find the optimal dose of HL156A in a rat UUO model. In that experiment, rats with UUO were given 0, 10, and 20 mg/kg of HL156A. Then, in the main animal experiment, rats were divided into four groups. The procedure of the UUO model has been well described elsewhere [23]. Briefly, adult male Wistar rats (7–8 weeks, body weight 180–200 g, Koatech Co, Seoul, Korea) were anesthetized via inhalation of 2–2.5% isoflurane. After flank incision, the left kidney and ureter were exposed, and the proximal ureter was ligated with a 4–0 silk. For the sham-operation group, the kidney and ureter were exposed, and then the skin was sutured without ureter ligation. Rats with UUO were administered HL156A (20mg/kg body weight per day) or vehicle via oral gavage one day before the operation and daily thereafter for 10 days. There were seven animals in each group. On day 10, the obstructed kidneys were harvested (Table 1). After the kidneys were hemi-sectioned, cortical portions were collected and frozen in liquid nitrogen for reverse transcription PCR (RT-PCR) or western blot analysis. For light microscopy, kidneys were fixed in 10% formalin at 4°C for 12–24 hours, processed in graded alcohol, embedded in paraffin blocks, and then stored at room temperature until analysis.

**Table 1:** Body weight parameters and kidney weight results 10 days after unilateral ureteral obstruction.

	Body weight (g)	Kidney weight (g)
Sham	213.4 ± 4.7	1.31 ± 0.03
Sham + HL156A	217.7 ± 2.2	1.33 ± 0.04
UUO	203.6 ± 4.7	3.87 ± 0.14
UUO + HL156A	210.3 ± 5.8	3.36 ± 0.34

Values are means ±SEs for seven animals for each group.

*p* <0.05 between UUO and Sham in kidney weight.

*p* <0.05 between UUO+HL156A and Sham in kidney weight.

### Histopathology

The degree of renal fibrosis was determined by Trichrome staining kit (860–031; Ventana, Medical Systems, Inc. Roche Diagnostic USA). For the evaluation of renal injury score, ten tubulointerstitial fields were randomly selected and examined in terms of tubular dilatation, tubular atrophy, and interstitial fibrosis. Renal injury score was semi-quantitatively calculated based on the percentage of involved area with an assigned value: 0, none; 1, <10%; 2, 10% to 25%; 3, 25% to 75%; and 4, >75% as described elsewhere [24].

Immunohistochemistry (IHC, Ventana Medical Systems, Inc) was performed as described in our previous study [22]. For the specific method, we followed the manufacture’s instruction. Paraffin tissue sections (4μm) were analyzed for immunohistochemistry using monoclonal antibodies against α-SMA (ab5694; Abcam, Cambridge, MA, USA), E-cadherin (sc-7870; Santa Cruz Biotechnology, CA, USA), fibronectin (ab6328; Abcam Cambridge, MA, USA) and Type IV collagen (sc-9301; Santa Cruz Biotechnology CA, USA).

Ten individual high-power fields (magnification, 200 x) per kidney were analyzed, and representative images were presented.

### Cell culture and experimental treatments

Normal rat kidney (NRK-52E) cells were cultured in DMEM containing 5% FBS at 37°C in a 5% CO_2_ atmosphere. Cells were treated with HL156A (30 μM) and TGF-β1 (10 ng/ml). Total RNA was extracted and subjected to reverse transcription PCR. Proteins extracted from total cell lysates were subjected to western blot analysis. All measurements were replicated at least three times.

### Reverse transcription-polymerase chain reaction (RT-PCR) analysis

TGF-β1, fibronectin, type IV collagen, Smad3, α-SMA, E-cadherin, and β-catenin gene expression were assessed using RT-PCR. Total RNA was isolated from kidney samples with TRIzol (Invitrogen Japan, Tokyo, Japan) using a guanidine thiocyanate extraction protocol. The concentration of total RNA was determined by measuring the optical density at 260 nm and the purity was checked as the 260 nm/280 nm ratio with expected values between 1.8 and 2.0. All samples were stored at -80°C until further analysis. Then, total RNA was reverse transcribed into cDNA and then subjected to RT- PCR using rat-specific primers. The following primers were used: TGF-β1, forward: TGTCCGGCAGCTGAAC, reverse: GGCTTCGCACCCACGTAG; fibronectin, forward: TGACAACTGCCGTAGACCTGG, reverse: TACTGGTTGTAGGAGTGGCCG; Type IV collagen, forward: TCCTTG TGACCAGGCATAGT, reverse TTGAACATCTCGCTCCTCTC; Smad3, forward: CCTGGGCAA GTTCTCCAGAG, reverse: CCATCGCTGGCATCTTCTGTG; α-SMA forward: GCTCTCTAAGGC GGCCTTTG, reverse: ACGAAGGAATAGCCACGCTCA, E-cadherin, forward: CAGGATTACAAG TTCCCGCCA, reverse: CACTGTCCGCTGCCTTCA; and β-catenin, forward: ACAGCACCT TCAGCACTCT, reverse: AAGTTCTTGGCTATTACGACA.

### Quantitative real-time PCR (qRT-PCR)

RNA was extracted from normal rat kidney (NRK-52E) cell line using TRIzol (Invitrogen Japan, Tokyo, Japan). For *in vivo*, Wistar rat tissue was homogenized by TRIzol. cDNA was obtained from the RNA by reverse transcription using a High Capacity cDNA Reverse Transcription Kit (Applied Biosystems Cheshire, UK). PCR was done using Power SYBR Green PCR Master Mix, and Quantstudio 3 Real time PCR System (Applied Biosystems, Foster City, CA, USA). The specific primer sequences for qRT-PCR are shown in the S1 Table.

### Western blot analysis

Using cellular fractionation NE-PER Nuclear Cytoplasmic Extraction Reagent kit (#78833; Thermo Scientific Pierce Rockford, IL, USA) and western blot analysis, we found that TFG-β1 treatment was sufficient to increase the fraction of p-Smad3 protein located in the nucleus. For this, 50 μg of protein was extracted from cell lysates, loaded into lanes, separated by 8% SDS-PAGE under reducing conditions, and transferred onto a nitrocellulose membrane (Amersham, Arlington Heights, IL, USA) by electro blotting. The protein transfer and volume consistency across lanes were verified using reversible staining with Ponceau S. The membrane was blocked with 5% nonfat dry milk in TBS-T (Tris-buffered saline and 0.15% Tween 20) for 1 hour at room temperature. Antibodies against the following proteins were used at 1:1,000 dilutions, unless otherwise described: TGF-β1 (sc-146; Santa Cruz Biotechnology CA, USA), α-SMA (ab5694; Abcam Cambridge, MA, USA), E-cadherin (sc-8426; Santa Cruz Biotechnology CA, USA), type IV collagen (sc-9301; Santa Cruz Biotechnology CA, USA), fibronectin (ab6328; Abcam Cambridge, MA, USA), Smad3 (#9513; Cell Signaling Technology, Inc., Danvers, MA, USA), MCP-1 (ab25124; Abcam Cambridge, MA, USA) and cleaved caspase 3 (#9661S Cell Signaling Technology, Inc., Danvers, MA, USA). The membranes were incubated overnight at 4°C in a 1:500 dilution of primary antibody. After three washes with TBS-T, the membrane was incubated with horseradish peroxidase-conjugated anti-mouse (sc-2020; Santa Cruz Biotechnology, CA, USA), and anti-rabbit Ig (sc-2005; Santa Cruz Biotechnology CA, USA) at a 1:2000 dilution, followed by enhanced chemiluminescence (ECL western blotting detection reagent, Amersham Biosciences). All the protein bands of western blot analysis were quantified using image J (Mac OS X; National Institute of Mental Health Bethesda, Maryland, USA) program and normalized to β-actin.

### Immunofluorescence staining and confocal microscopy

NRK-52E cells cultured on coverslips were fixed with 4% PFA for 15 minutes at RT. After blocking with 1% BSA for 30 minutes, the slides were immunostained with primary antibodies for p-AMPK (sc-33524; Santa Cruz Biotechnology CA, USA), p-Smad3 (ab52903; Abcam Cambridge, MA, USA), E-cadherin, (sc-8426; Santa Cruz Biotechnology CA, USA), α-SMA (ab5694; Abcam Cambridge, MA, USA), fibronectin (sc-9068; Santa Cruz Biotechnology CA, USA), and type IV collagen (sc-9301; Santa Cruz Biotechnology CA, USA). Next, the slides were stained with a secondary antibody (Alexa) and mounted with mounting media (Dako, San Diego, CA, USA) using 4, 6-diamidino-2-phenylindole to visualize the nuclei (Sigma Aldrich). Slides were viewed under a Leica TSL-SL confocal microscope. Staining intensity was measured using Image J analysis software ((Mac OS X; National Institute of Mental Health Bethesda, Maryland, USA)

### Statistical analyses

Statistical analyses were performed using the Analysis of Variance (ANOVA) test, and *post-hoc* analyses were performed using Tukey’s HSD test. P-values <0.05 were considered statistically significant.

## Results

### AMPK activation by AICAR, metformin, and HL156A

**Fig 1:**
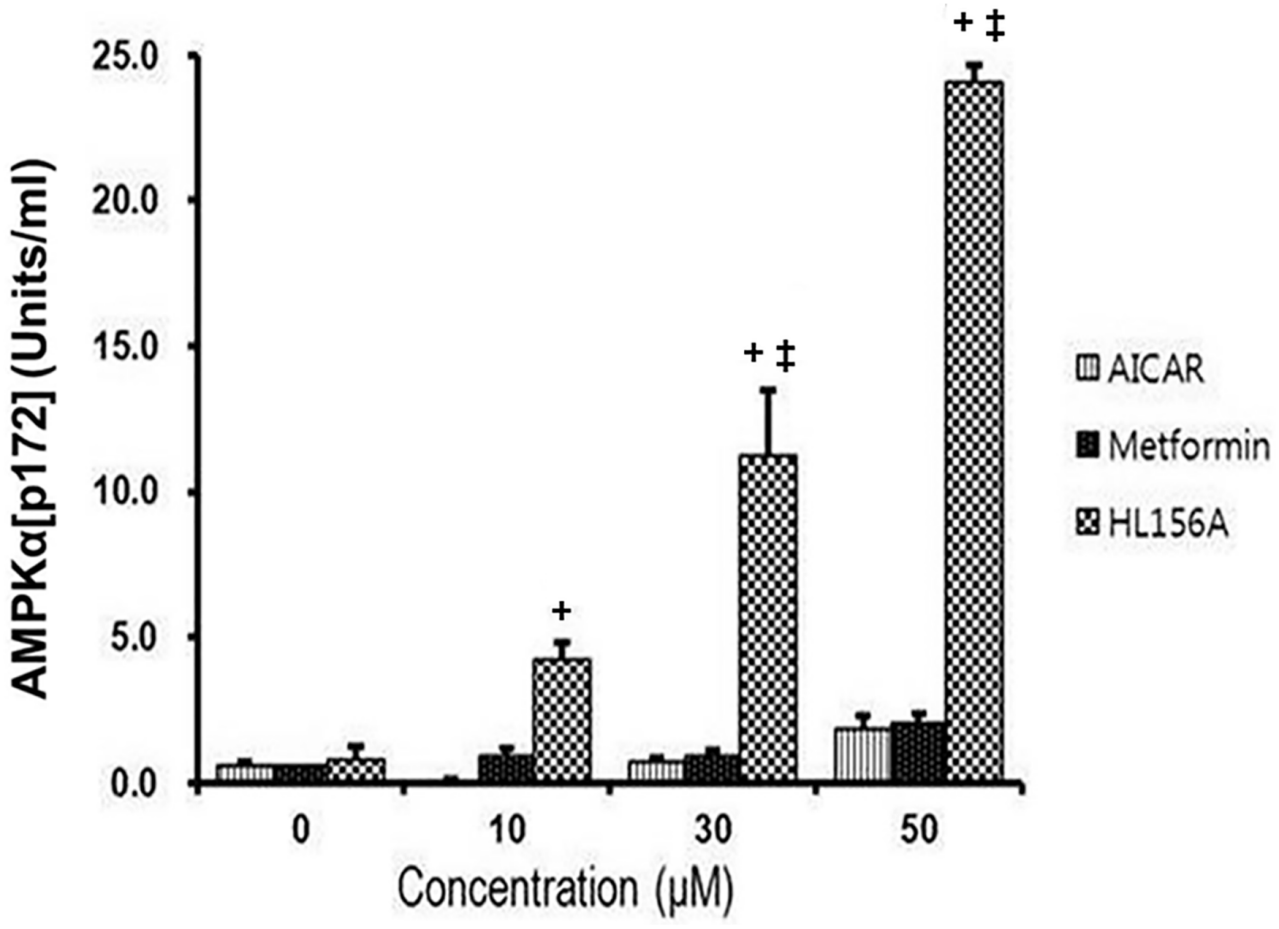
Comparison of AMPK activator activities. AMPK activities induced by various AMPK activator molecules were determined on rat renal proximal tubular (NRK-52E) cells. The AMPK activator activities of 5-aminoimidazole-4-carboxamide-1-β-D-ribofuranoside (AICAR), metformin, and HL156A were compared at various concentrations (0, 10, 30, and 50 μM, respectively). AMPK activator activity was determined by AMPKαThr172 phosphorylation and was measured using an ELISA kit (Invitrogen Corporation). Experiments were repeated at three times. ^+^: P <0.05 vs. AICAR, ^**‡**^: P <0.05 vs. metformin at the same concentration.

NRK-52E cells were supplemented with various concentrations (0, 10, 30, and 50 μM, respectively) of AICAR, metformin and HL156A for 24 hours. Then, AMPK-α phosphorylation at Thr172 was determined by ELISA kit (KHO0651; Invitrogen Corporation, Camarillo, CA, USA) at each concentration of various AMPK activators. At 10 μM concentration of each AMPK activator, the degree of AMPK-α phosphorylation by HL156A was significantly higher than that by AICAR. At 30 and 50 μM concentrations, AMPK-α phosphorylation by HL156A was significantly more potent than by AICAR and metformin (Fig 1).

### Preliminary study: HL156A dose finding experiment

**Fig 2:**
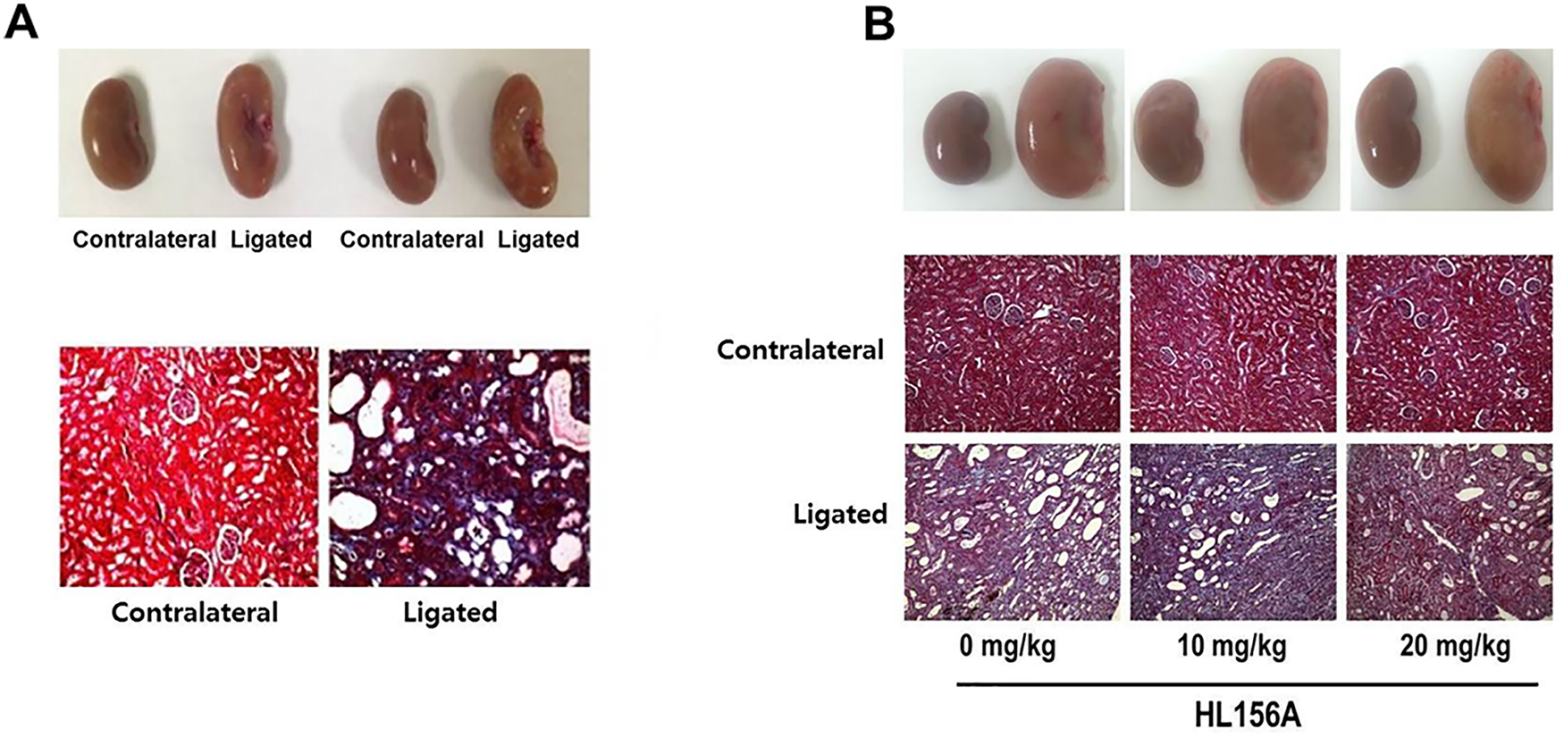
The effect of HL156A treatment on UUO. In a preliminary experiment, a unilateral ureteral obstruction (UUO) model was induced in adult male Wistar rats. Obstructed kidneys were harvested 10 days after surgery. (A) Each ligated kidney was compared with its contralateral (non-ligated) kidney after Masson’s trichrome staining. (B) To find the optimal dose of HL156A, the rats were subjected to unilateral ureter ligation and then treated with various doses of HL156A (0, 10, and 20 mg/kg/day for 10 days) before comparative analyses were performed. Experiments were repeated at two times with N = 4 each time. Treatment with 20mg/kg of HL156A markedly ameliorated the renal injury from UUO.

Preliminary experiment confirmed that the rats with unilateral ureter ligation showed significantly increased tubular dilatation, tubular necrosis and interstitial fibrosis of the cortex and medulla, compared to the contralateral kidney (Fig 2A). Treatment with 20mg/kg of HL156A markedly ameliorated the renal injury from UUO (Fig 2B).

### HL156A ameliorated histological changes in UUO-induced renal injury

**Fig 3:**
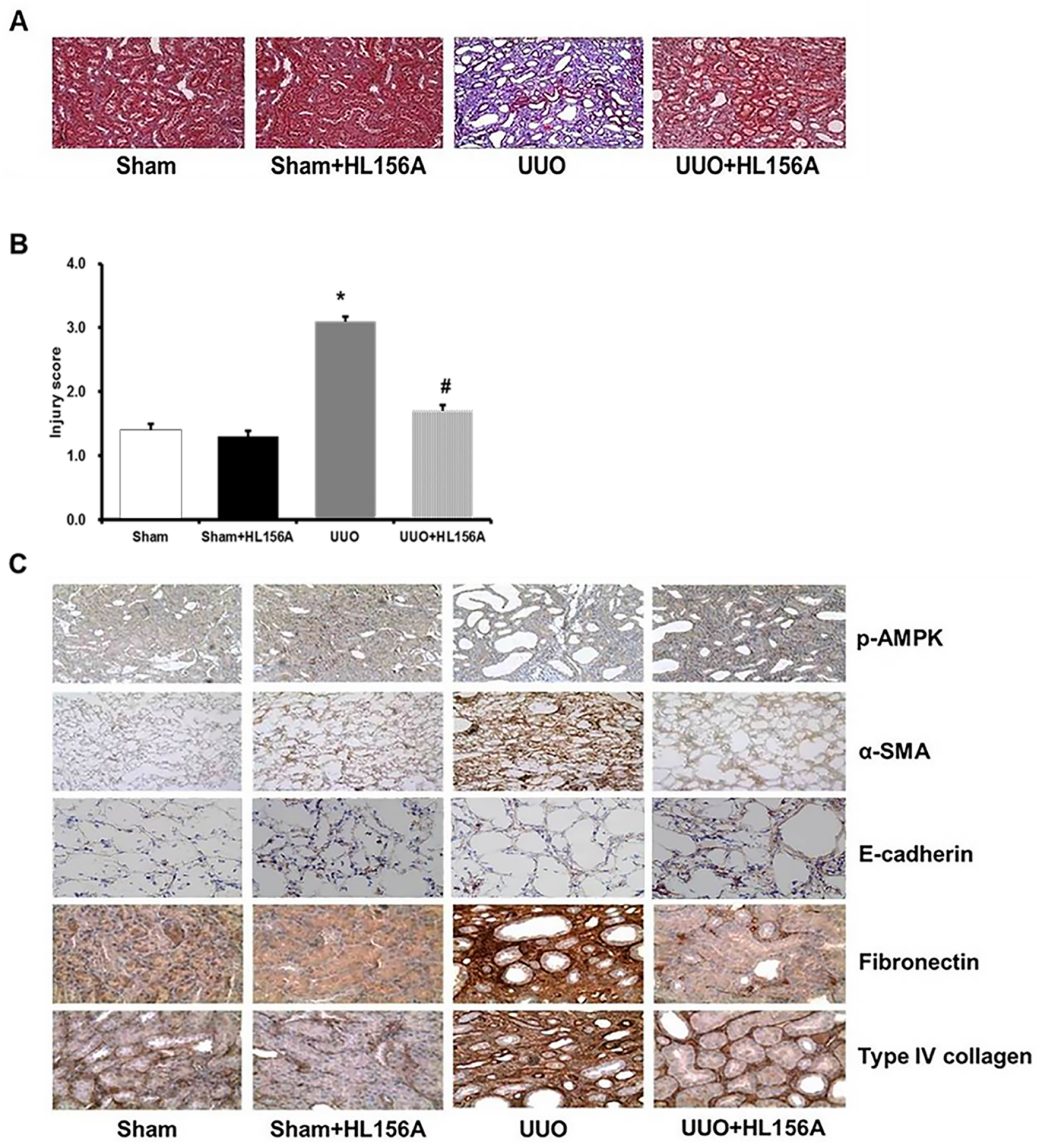
HL156A ameliorated histopathological changes associated with UUO. A unilateral ureteral obstruction (UUO) model was induced in adult male Wistar rats. Rats with UUO were administered HL156A (20 mg/kg/day) or saline one day before the ligation surgery and daily thereafter. Obstructed kidneys were harvested 10 days after surgery. (A) Masson’s trichrome shows that UUO induced severe renal fibrosis, which was significantly ameliorated by HL156A. (B) Quantification of renal injury score based on the Masson’s trichrome staining of the kidneys. (C) Immunohistochemical staining shows UUO down-regulated p-AMPK, thereby inducing an increase in α-SMA and a down-regulation of E-cadherin, both of which are hallmarks of EMT. UUO increased expression of extracellular matrix (ECM) molecules, such as fibronectin and type IV collagen. HL156A treatment, which recovered down-regulated p-AMPK, significantly diminished the above changes induced by UUO. Experiments were repeated at two times with N = 7 each time. * P <0.05 *vs*. Sham, ^#^P <0.05 between UUO and UUO + HL156A.

Ureteral ligation induced severe renal tubular damage and interstitial fibrosis (Fig 3A and 3B), which was associated with a marked reduction of p-AMPKα (Fig 3C). Immunohistochemistry showed an increase of α-SMA with down-regulation of E-cadherin, both of which are hallmarks of EMT. UUO groups also exhibited increased expression of extracellular matrix (ECM) molecules, including fibronectin and type IV collagen (Fig 3C). Compared with the vehicle-treated UUO group, the HL156A-treated UUO group exhibited marked amelioration of renal injury score (Fig 3A and 3B), markedly increased p-AMPKα expression, and reduced expression of α-SMA, fibronectin, and type IV collagen. E-cadherin expression was also restored in the renal tubules of the obstructed kidney by HL156A treatment (Fig 3C).

### HL156A ameliorated the fibrogenic signals induced by UUO

**Fig 4:**
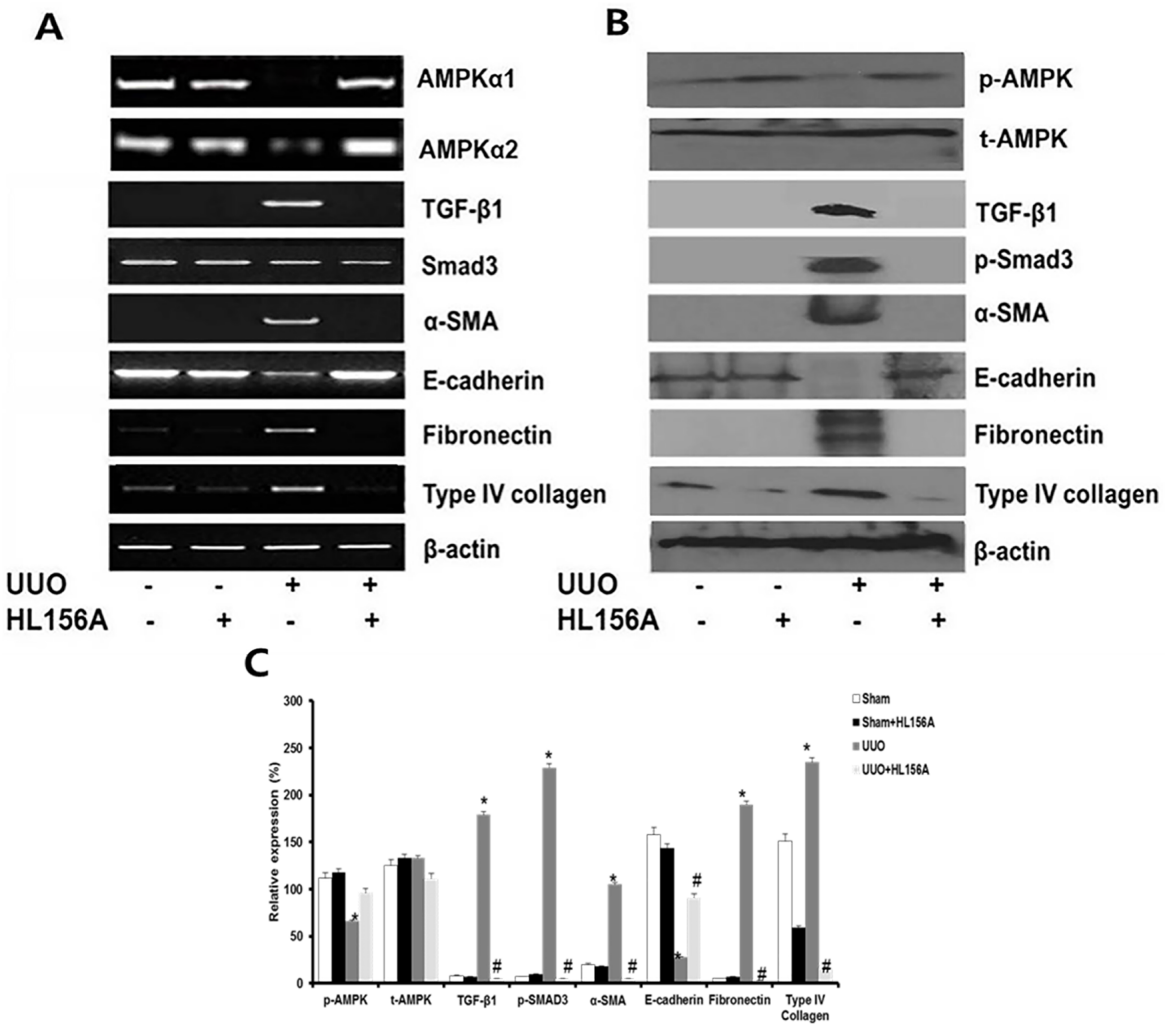
Effects of HL156A treatment on mRNA and protein expression. mRNA and protein expressions of various markers were analyzed from the animal experiment. (A) Reverse transcription PCR was used to analyze mRNA expression levels of AMPKα1, AMPKα2, TGF- β1, Smad3, α-SMA, fibronectin, E-cadherin, and type IV collagen. HL156A treatment recovered the down-regulations of AMPKα1 and AMPKα2 induced by UUO. (B) Western blot analysis was used to measure phosphorylated AMPKα, total AMPK, TGF- β1, Smad3, α-SMA, fibronectin, E-cadherin, and type IV collagen expression levels from kidney tissue. (C) Western blot analysis of protein bands quantified using image J and normalized to β-actin. Experiments were repeated two times with N = 7 at each time. *P <0.05 vs. sham, ^#^P <0.05 between UUO and UUO + HL156A.

Next, we confirmed the expression of EMT markers and fibrogenic signals in the kidney tissues by RT-PCR and western blot. Compared with the sham-operated kidneys, kidneys from the UUO group exhibited significant down-regulation of AMPKα1 and AMPKα2 and up-regulation of TGF-β1, p-Smad3, α-SMA, fibronectin, and type IV collagen genes. Western blot showed changes in p-AMPK, TGF-β1, p-Smad3, α-SMA, fibronectin, and type IV collagen molecules that were similar to those observed in the RT-PCR results. HL156A treatment restored AMPK and above-mentioned fibrogenic signals both at the mRNA and protein levels (Fig 4). E-cadherin expression was markedly decreased in the UUO animals, which expression was recovered by HL156A treatment. Similar changes were also shown in qRT-PCR results (S1 Fig).

### Optimal dose and exposure time of TGF-β1 in a cultured rat proximal cell line

**Fig 5:**
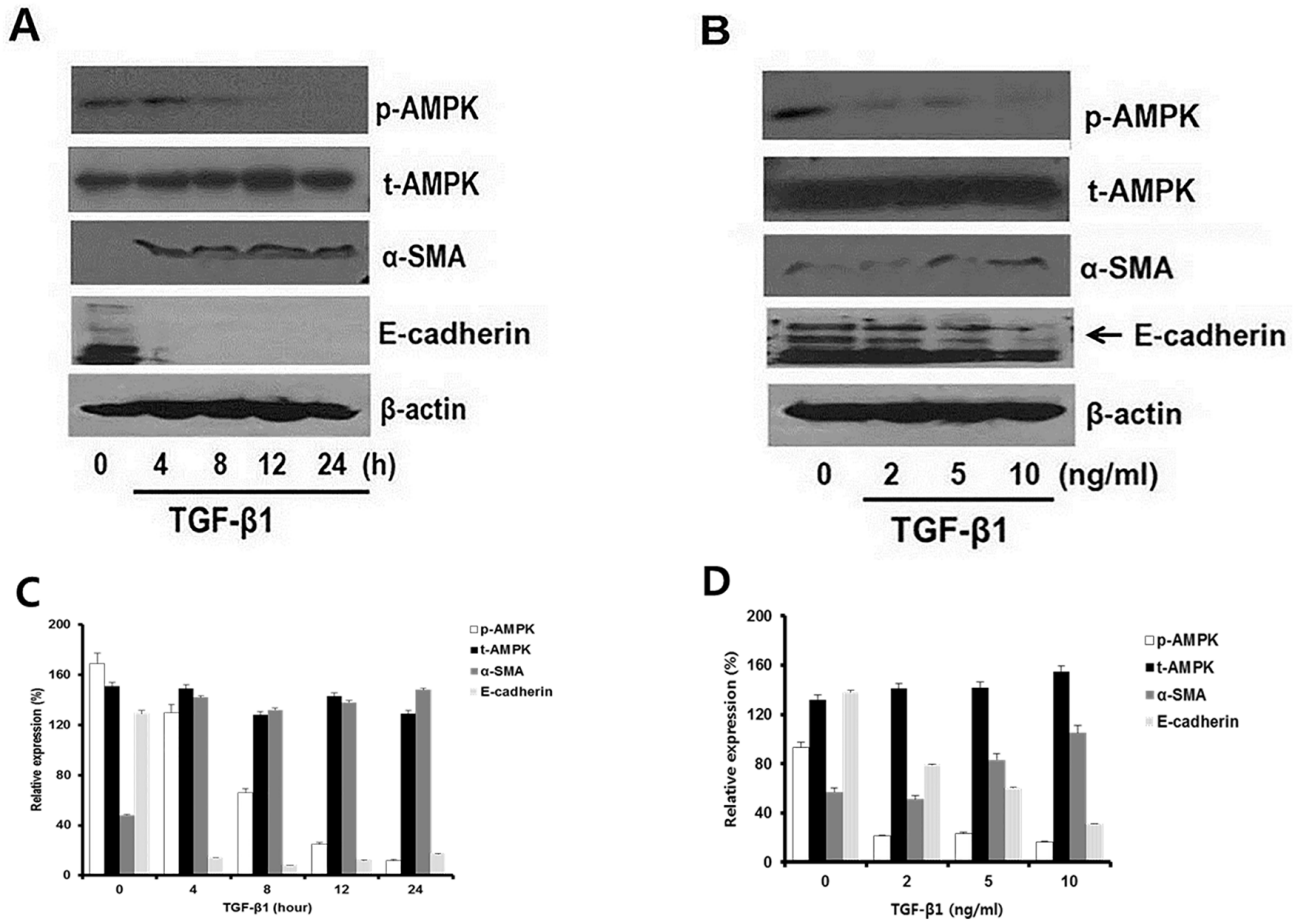
Pilot study to estimate the optimal dose and duration of TGF-β1 treatment. Cultured rat proximal tubular NRK-52E cells were incubated in the different concentrations TGF-β1 and various exposure time. (A) NRK-52E cells were treated with TGF-β1 at different concentrations (0, 2, 5, and 10 ng/ml) for 24 hours and were subjected to western blot analysis to evaluate phosphorylation of AMPK, total AMPK, α-SMA, E-cadherin, and β-actin. (B) NRK-52E cells were exposed to TGF-β1 at various exposure times (0, 4, 8, 12, and 24 hours), and AMPK, α-SMA, and E-cadherin expressions were evaluated. The results showed that 10 ng/ml of TGF-β1 for 24 hours were the optimal dose and time for our further experiments. (C, D) Western blot analysis quantified protein bands by using image J and normalized to β-actin. Experiments were repeated three times.

We further explored whether the above changes in renal tissues induced by AMPK activation were reproducible in rat proximal tubule cells. First, since TGF-β1 is a key player in the EMT and in renal fibrosis, we explored the optimal TGF-β1 dose and exposure time from cultured NRK52E cells (rat proximal cell line). There was a dose-dependent and time-dependent change in p-AMPK, α-SMA, and E-cadherin in response to TGF-β1 exposure. We finally concluded that 10 ng/ml of TGF-β1 and a 24-hour exposure were optimal for the *in vitro* experiment (Fig 5).

### HL156A up-regulated AMPK and inhibited fibrogenic signals from NRK52E cells

**Fig 6:**
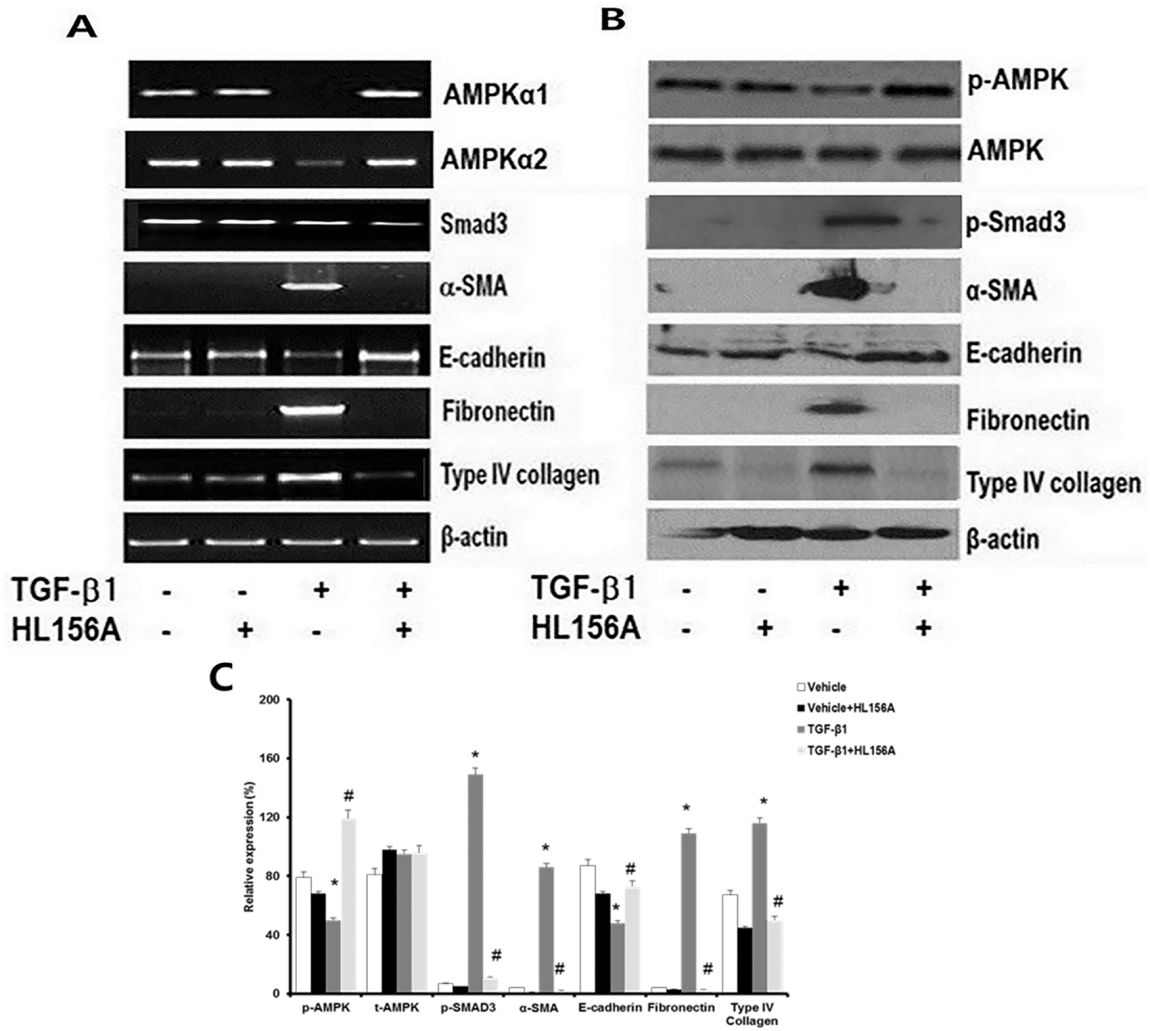
The effect of HL156A on TGF-β1-treated NRK52E cells. NRK-52E cells, cultured rat proximal cells, were incubated in the presence or absence of HL156A 30 μM for 24 hours. Then, these cells were stimulated with TGF-β1 (10 ng/mL) for 24 hours before harvesting. Compared with TGF-β1 treatment alone, (A) HL156A co-treatment up-regulated AMPKα1, AMPKα2, and E-cadherin expressions. HL156A down-regulated Smad3, α-SMA, fibronectin, and type IV collagen. This was confirmed by mRNA expression levels of AMPKα1, AMPKα2, TGF-β1, Smad3, α-SMA, fibronectin, E-cadherin, and type IV collagen. (B) Representative Western blots of phosphorylated AMPKα, total AMPK TGF-β1, Smad3, α-SMA, fibronectin, E-cadherin, and type IV collagen expression levels from the NRK-52E cells. (C) Western blot analysis of protein bands quantified using image J and normalized to β-actin. Experiments were repeated three times. *P <0.05 vs. vehicle, ^#^P < 0.05 between TGF-β1 and TGF-β1 + HL156A.

The effect of HL156A on the TGF-β1-treated NRK52E cell line was evaluated. In RT-PCR, compared with TGF-β1 treatment alone, HL156A co-treatment up-regulated the transcription levels of AMPKα1, AMPKα2, and E-cadherin expression and down-regulated Smad3, α-SMA, fibronectin, and type IV collagen expression (Fig 6A). Western blot demonstrated that HL156A treatment stimulated p-AMPK and inhibited the TGF-β1-induced phosphorylation of Smad3 and expression of its downstream molecules in the canonical TGF-β1/Smad3 pathway from NRK52E cells (Fig 6B and 6C). The anti-fibrogenic effects of HL156A *in vitro* were also shown in our qRT-PCR results (S2 Fig).

### HL156A ameliorated the apoptotic signal *in vivo* and *in vitro*

We analyzed cleaved caspase 3 as an apoptotic signal from the renal tissues by western blots. Obstructed kidneys from UUO rat exhibited increased expression of cleaved caspase 3, which was markedly ameliorated by HL156A treatment (S3 Fig). This protective effect of HL156A against apoptosis was also shown in TGF-β-treated NRK-52E cells (S3 Fig).

### The expression of inflammatory chemokine

**Fig 7:**
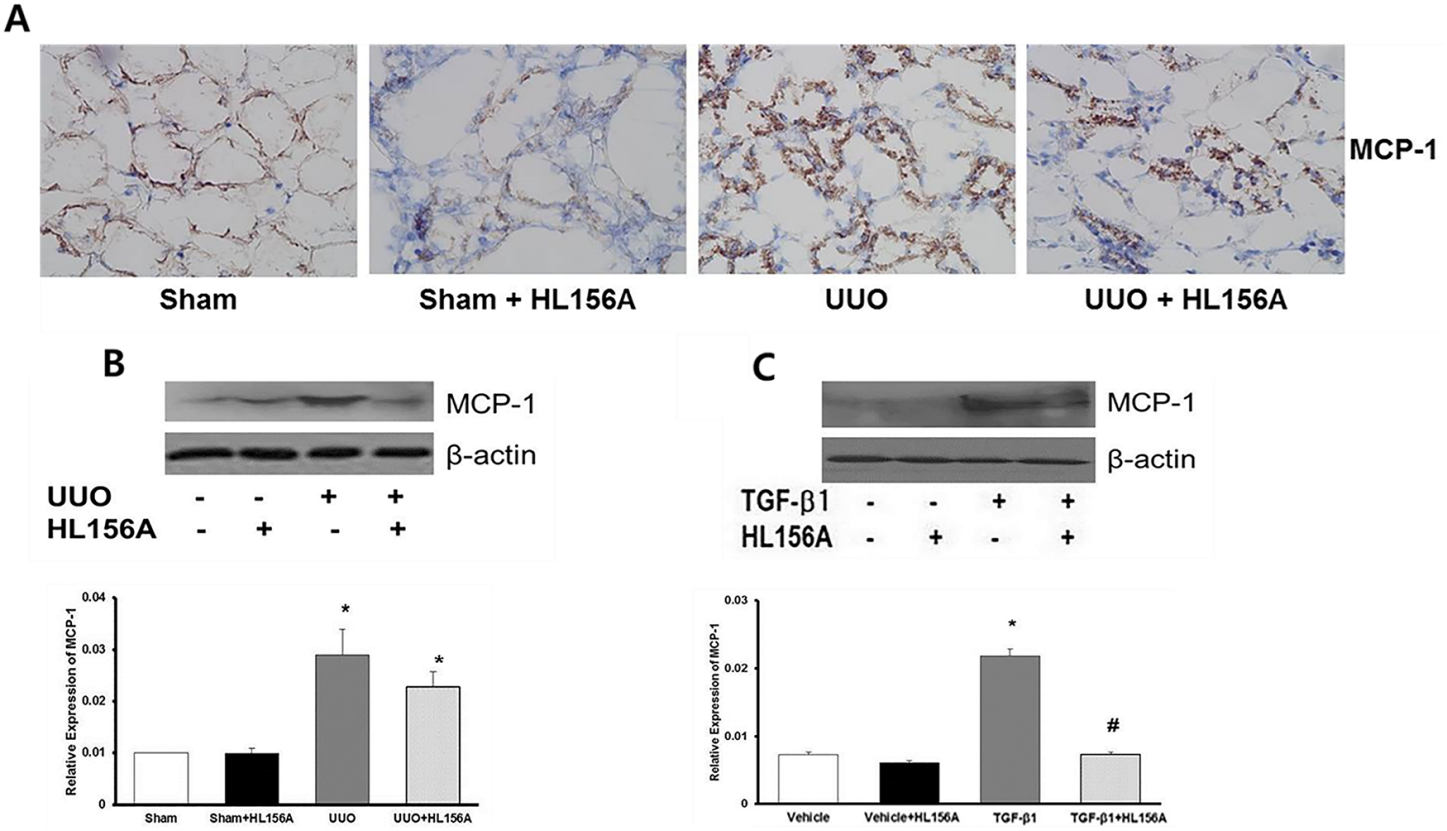
The expression of inflammatory cytokine. (A) Immunohistochemistry (IHC) staining showed expression of MCP-1 (brown). This revealed that HL156A inhibited MCP-1, which was induced by UUO group. (B) qRT-PCR and western blot data showed that HL156A treatment improved the MCP-1 under the UUO group. (C) HL156A treatment induced of MCP-1 from NRK52E cells. Experiments were repeated two times. *P <0.05 vs. Sham or vehicle, ^#^P < 0.05 between TGF-β1 and TGF-β1 + HL156A.

Immunohistochemistry analysis revealed that HL156A inhibited the UUO induced MCP-1 expression in rat kidney (Fig 7A). qRT-PCR and western blot analysis demonstrated that HL156A treatment down-regulated the MCP-1 expression from the kidney tissues (Fig 7B) and from the NRK52E cells (Fig 7C). These results indicate that HL156A inhibits the expression of inflammatory signals in the rat kidney and renal proximal tubule cell line.

### Immunofluorescence analysis of AMPK and the Smad3 pathway

**Fig 8:**
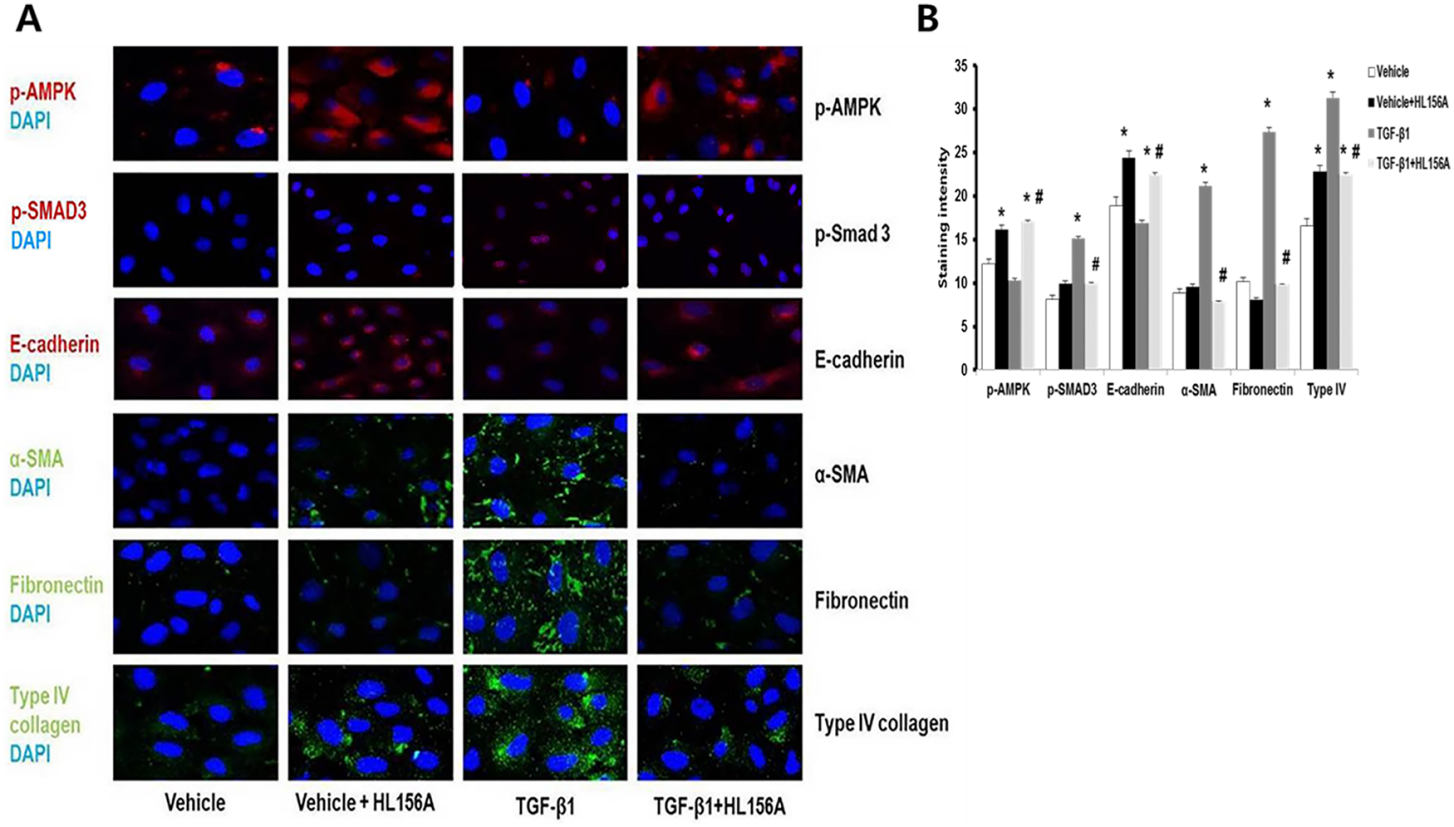
Immunofluorescence analysis of AMPK and Smad3 pathways. NRK52E cells were treated with TGF-β1 (10 ng/ml) with or without HL156A (30 μM) for 24 hours. (A) Immunofluorescence staining showed expression of p-AMPK (red), p-Smad3 (red), E-cadherin (red), α-SMA (green), fibronectin (green), and type IV collagen (green). This revealed that HL156A, by stimulating p-AMPK, inhibited p-Smad3, α-SMA, fibronectin, and type IV collagen, which were all induced by TGF-β1 treatment. HL156A co-treatment also restored E-cadherin expression that was suppressed under TGF-β1 treatment. (B) Immunofluorescence analysis quantified using image J. Experiments were repeated three times. *P <0.05 vs. Vehicle, ^#^P < 0.05 between TGF-β1 and TGF-β1 + HL156A.

Immunofluorescence analysis revealed that HL156A, by stimulating p-AMPK, inhibited the TGF-β1-induced p-Smad3, α-SMA, fibronectin, and type IV collagen in NRK52E cells. HL156A also abrogated the down-regulation of E-cadherin under the TGF-β1-treated condition (Fig 8). Taken together, these results indicate that HL156A down-regulated EMT markers and inhibited ECM expression by inhibiting p-Smad3 and downstream molecules in the renal proximal tubule cell line.

## Discussion

The purpose of this study was to evaluate the amelioration of renal fibrosis by HL156A, a novel AMPK activator molecule, in a rat UUO model. In a previous report, we showed that HL156A activated AMPK, resulting in amelioration of peritoneal fibrosis [22]. The present study demonstrated that HL156A treatment attenuated tubulointerstitial fibrosis in the obstructed kidney of a rodent UUO model. We compared the AMPK-activator activities of AICAR, metformin, and HL156A in a rodent renal tubular epithelial cell line. Among the three AMPK activator substances, AMPK activation was the most potent in the presence of the HL156A molecule. We showed that HL156A reduced renal fibrosis and ECM expression. From *in vitro* experiments, we found that HL156A decreased α-SMA expression and recovered E-cadherin expression in TGF- β1 treated cells.

AMPK is a metabolic regulator protein that is activated by nutrient and bioenergetic stress, which rapidly consumes ATP and elevates intracellular AMP. AMPK, when activated, switches off the energy-consuming pathways and activates the energy-producing pathways, resulting in restoration of intracellular energy homeostasis. Recent reports have shown novel functions associated with AMPK beyond metabolic regulation, including anti-fibrotic and anti-tumor effects[25], [26]. AMPK is strongly expressed in the kidneys, where it is involved in diverse physiologic and pathologic processes[27]. In light of this, we investigated the inhibitory effects of HL156A, which demonstrated more potent AMPK activity on renal fibrosis than AICAR or metformin in a rat UUO model. TGF-β1, a key molecule of the TGF-β1 superfamily, plays a pivotal role in the activation of inflammatory cells[28], in the EMT, and in fibrosis development[29]. TGF-β1 exerts its biological and pathological activity by binding to its receptors and signaling in Smad-dependent and independent pathways[30]. A previous study demonstrated that metformin activates AMPK and, thus, suppresses collagen type I production via renal fibroblasts in response to TGF-β1 [31]. Metformin and AICAR have been shown to activate AMPK in several organs[32], and recent studies have shown that these AMPK activators have a protective effect against renal fibrosis in animal models. AICAR reduced tubulointerstitial fibrosis in UUO mice and inhibited TGF-β1-induced myofibroblast activation [33], [34]. Our study demonstrated that AMPK activation by HL156A treatment resulted in decreased renal fibrosis in rats with UUO. We showed that HL156A ameliorated the signals associated with EMT and ECM expression in both *in vivo* and *in vitro* experiments. In our *in vitro* experiment, such protective effects were associated with inhibition of the Smad3 phosphorylation and nuclear translocation in rat proximal tubule cells. This is in contrast with a report by Mishra et al.[35], who showed that AMPK did not reduce Smad3 phosphorylation or nuclear translocation in TGF-β1-stimulated human primary mesangial cells. It might be that the specific inhibitory effect of AMPK on TGF-β1 signaling is context-dependent on the species and cell type.

Collectively, the major finding of our study is that HL156A, a novel AMPK activator, elicits beneficial effects on renal fibrosis and restoration from EMT. Further studies are needed to investigate the intracellular link between AMPK and signaling cascades downstream of TGF-β1, such as Smad-dependent and Smad-independent pathways of tissue fibrosis.

In conclusion, we demonstrated that HL156A, a novel AMPK activator, reduced tubulointerstitial fibrosis in UUO rats *in vivo* and inhibited signaling cascades downstream of p-Smad3 after TGF-β1-stimulation *in vitro*.

## Supporting information

S1 Table Primer sequences for quantitative real time PCR. (DOCX)

S1 Fig Quantitative real-time PCR for various gene expressions in the kidney tissue. (DOCX)

S2 Fig Quantitative real-time PCR for various gene expressions in the NRK52E cells. (DOCX)

S3 Fig Cleaved caspase 3 expression *in vivo* and *in vitro*. (DOCX)
