# Supplementary material for: HL156A, a novel pharmacological agent with potent adenosine-monophosphate-activated protein kinase (AMPK) activator activity ameliorates renal fibrosis in a rat unilateral ureteral obstruction model
Source: PLoS One. 2018 Aug 30;13(8):e0201692. doi: 10.1371/journal.pone.0201692 (PMC6116936; doi:10.1371/journal.pone.0201692)
Supplement: S1 Table — (DOCX) [file pone.0201692.s001.docx]

**Supporting Information**

**S1 Table. Primer sequences for quantitative real time PCR**

| **Expressed genes** | **Forward Sequence** | **Reverse Sequence** |
| --- | --- | --- |
| AMPKα1 | 5’CTCAGTTCCTGGAGA AAG ATGG3’ | 5’CTGCCGGTTGAGTATCTTCAC3’ |
| AMPKα2 | 5’GCTGTGGATCGCCAA ATTAT3’ | 5’GCATCAGCAGAGTGGCAATA3’ |
| TGF-β1 | 5’TATAGCAACAATTCCTGGCGTTAC3’ | 5’TGTATTCCGTCTCCTTGGTCA3’ |
| Smad3 | 5’GGTAAAGGATTGCCACCA AA3’ | 5’GAACAGCCAGGAAAGGGACT3’ |
| α-SMA | 5’GCTCTCTAAGGCGGCCTTTG3’ | 5’ACGAAGGAATAGCCACGCTCA3’ |
| E-cadherin | 5’GCCCAGGAGCTGACA AAC3’ | 5’CCAGAGGCTGCGTCACTTTC3’ |
| Fibronectin | 5’TGACAACTGCCGTAGACCTGG3’ | 5’TACTGGTTGTAGGAGTGG CCG3’ |
| Type IV Collagen | 5’TGTAACGTCGATAACGTGTGT3’ | 5’TGCTAACGTAGG GCTCAAGG3’ |
| MCP-1 | 5’CCAGTGGCTCTTTCCCTGAG3’ | 5’CACAGGTGGCTTGGCTATGA3’ |
| β-catenin | 5’ACAGCA CCTTCA GCA CTCT3’ | 5’AAGTTCTTGGCTATTACGACA3’ |
