# Supplementary material for: HL156A, a novel pharmacological agent with potent adenosine-monophosphate-activated protein kinase (AMPK) activator activity ameliorates renal fibrosis in a rat unilateral ureteral obstruction model
Source: PLoS One. 2018 Aug 30;13(8):e0201692. doi: 10.1371/journal.pone.0201692 (PMC6116936; doi:10.1371/journal.pone.0201692)
Supplement: S1 Fig — (DOCX) [file pone.0201692.s002.docx]

**S1 Fig. Quantitative real-time PCR for various gene expressions in the kidney tissue**

**
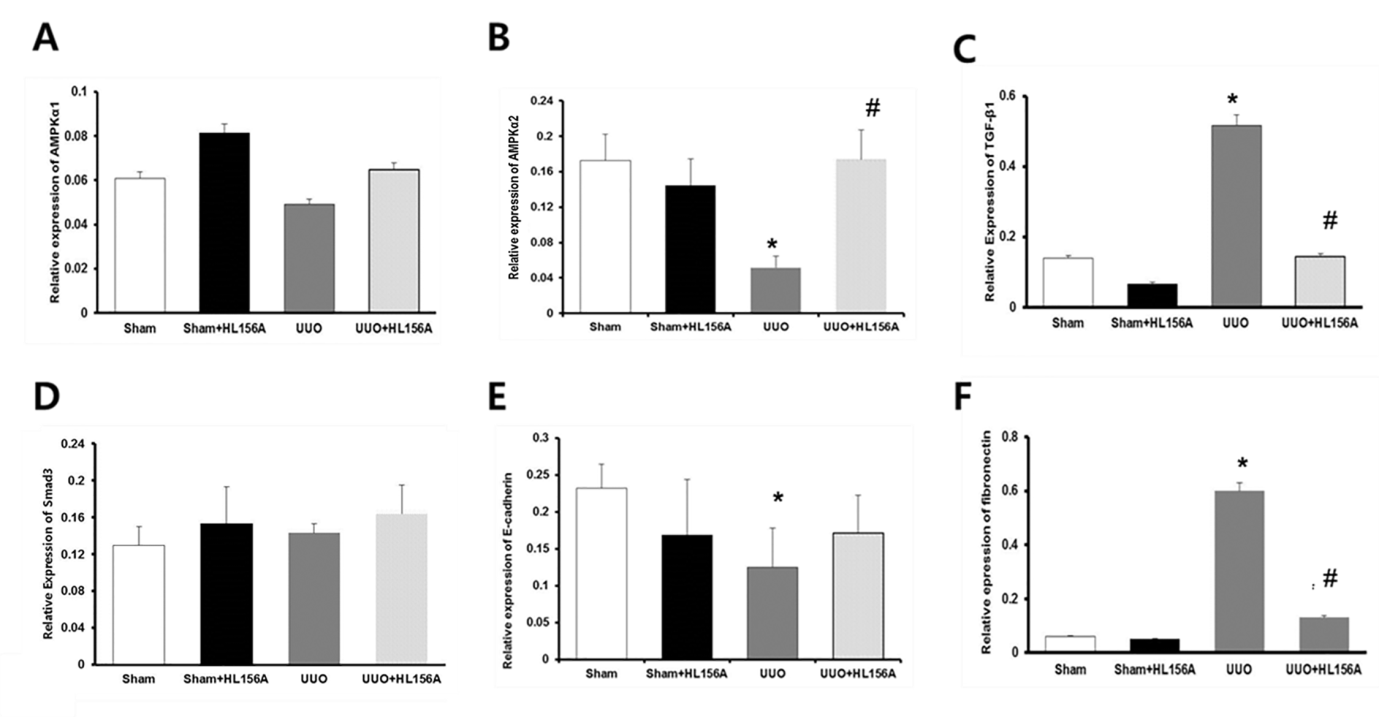
**

RNA was extracted from Wistar rat kidney using TRIzol (Invitrogen Japan, Tokyo, Japan). The concentration of total RNA was determined by measuring the optical density at 260 nm and the purity was checked as the 260 nm/280 nm ratio with expected values between 1.8 and 2.0. cDNA was obtained from the RNA by reverse transcription using a High Capacity cDNA Reverse Transcription Kit (Applied Biosystems Cheshire, UK). Primers were used and all samples were stored at -80°C until further analysis. PCR was done using Power SYBR Green PCR Master Mix and Quantstudio 3 Real time PCR System (Applied Biosystems). Results are presented by ΔCt. Compared with unilateral ureteral obstruction (UUO) alone, HL156A co-treatment up-regulated AMPKα1 (A), AMPKα2 (B), and E-cadherin (E) expressions. HL156A down-regulated TGF-β (C) and fibronectin (F) expression. Smad3 mRNA expression was similar between the four groups (D). *P <0.05 vs. Sham, #P <0.05 vs. UUO. This was performed twice and each experiment included two animals from each group.
