# Supplementary material for: HL156A, a novel pharmacological agent with potent adenosine-monophosphate-activated protein kinase (AMPK) activator activity ameliorates renal fibrosis in a rat unilateral ureteral obstruction model
Source: PLoS One. 2018 Aug 30;13(8):e0201692. doi: 10.1371/journal.pone.0201692 (PMC6116936; doi:10.1371/journal.pone.0201692)
Supplement: S2 Fig — (DOCX) [file pone.0201692.s003.docx]

**S2 Fig. Quantitative real-time PCR for various gene expressions in the NRK52E cells**

**
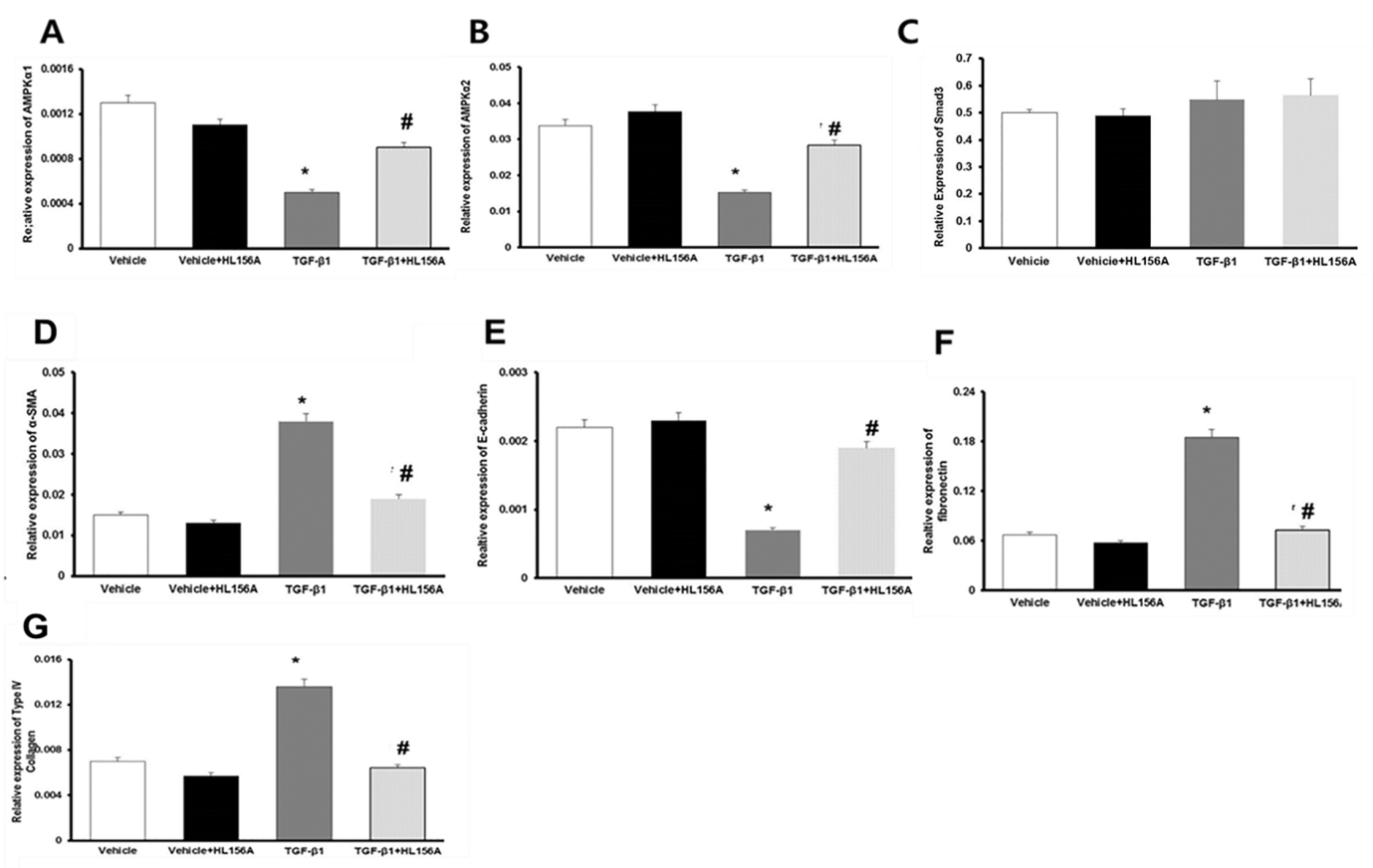
**

RNA was extracted normal rat kidney (NRK-52E) cell line using TRIzol (Invitrogen Japan, Tokyo, Japan). cDNA was obtained from the RNA by reverse transcription using a High Capacity cDNA Reverse Transcription Kit (Applied Biosystems Cheshire, UK). PCR was done using Power SYBR Green PCR Master Mix and Quantstudio 3 Real time PCR System (Applied Biosystems). Results are presented by ΔCt. qRT-PCR showed an increase of α-SMA (D) with down-regulation of E-cadherin (E), both of which are hallmarks of EMT. TGF-β1 groups also exhibited increased expression of extracellular matrix (ECM) molecules, including fibronectin (F), type IV collagen (G). Smad3 mRNA expression was not different among the four groups (C). Compared with the TGF-β1 group, the HL156A-treated TGF-β1 group exhibited markedly increased AMPKα1 (A) and AMPKα2 (B) expression and reduced expression of α-SMA, fibronectin, and type IV collagen. E-cadherin expression was also restored in the HL156A treatment group. *P <0.05 vs. Control (CTR), #P <0.05 vs. TGF-β1. Each experiment was performed twice.
