# Supplementary material for: HL156A, a novel pharmacological agent with potent adenosine-monophosphate-activated protein kinase (AMPK) activator activity ameliorates renal fibrosis in a rat unilateral ureteral obstruction model
Source: PLoS One. 2018 Aug 30;13(8):e0201692. doi: 10.1371/journal.pone.0201692 (PMC6116936; doi:10.1371/journal.pone.0201692)
Supplement: S3 Fig — (DOCX) [file pone.0201692.s004.docx]

**S3 Fig. Cleaved caspase 3 expression *in vivo* and *in vitro***


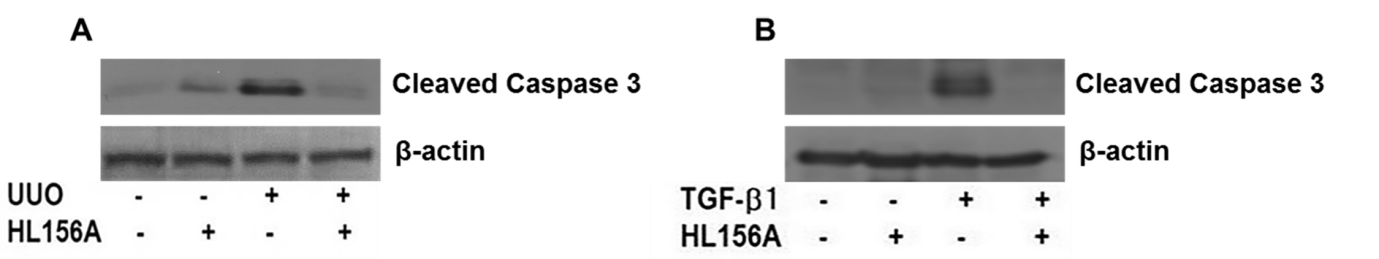


Effect of HL156A on UUO-induced cell apoptosis in the obstructed kidney was analyzed. Representative western blots of cleaved caspase 3 (#9661S Cell signaling Technology, Inc., Danvers, MA, USA) protein from the obstructed rat kidneys. (A) Cleaved caspase 3 expression was increased in obstructed kidneys from UUO rat, and HL156A reduced the expression of cleaved caspase 3. Representative western blots of cleaved caspase 3 from the TGF-β1–induced NRK-52E cells. (B) The protein expression of cleaved caspase 3 was significantly increased by TGF-β. HL156A treatment reversed above changes in the NRK-52E cells. Each experiment was performed twice.
